# Sodium Energetic Cycle in the Natronophilic Bacterium *Thioalkalivibrio versutus*

**DOI:** 10.3390/ijms23041965

**Published:** 2022-02-10

**Authors:** Maria S. Muntyan, Mikhail B. Viryasov, Dimitry Y. Sorokin, Vladimir P. Skulachev

**Affiliations:** 1 Belozersky Institute of Physico-Chemical Biology, Lomonosov Moscow State University, Leninskie Gory, 119991 Moscow, Russia; viryasov@genebee.msu.ru (M.B.V.); skulach@genebee.msu.ru (V.P.S.); 2 Winogradsky Institute of Microbiology, Federal Research Centre of Biotechnology, Russian Academy of Sciences, 117312 Moscow, Russia; soroc@inmi.ru; 3 Department of Biotechnology, Delft University of Technology, 2628 BC Delft, The Netherlands

**Keywords:** flagellar motor, Na^+^-motive cytochrome oxidase, alkaliphiles, Na^+^ energetic cycle, sodium-motive force

## Abstract

As inhabitants of soda lakes, *Thioalkalivibrio versutus* are halo- and alkaliphilic bacteria that have previously been shown to respire with the first demonstrated Na^+^-translocating cytochrome-*c* oxidase (CO). The enzyme generates a sodium-motive force (Δ*s*) as high as −270 mV across the bacterial plasma membrane. However, in these bacteria, operation of the possible Δ*s* consumers has not been proven. We obtained motile cells and used them to study the supposed Na^+^ energetic cycle in these bacteria. The resulting motility was activated in the presence of the protonophore 2-heptyl-4-hydroxyquinoline N-oxide (HQNO), in line with the same effect on cell respiration, and was fully blocked by amiloride—an inhibitor of Na^+^-motive flagella. In immotile starving bacteria, ascorbate triggered CO-mediated respiration and motility, both showing the same dependence on sodium concentration. We concluded that, in *T. versutus*, Na^+^-translocating CO and Na^+^-motive flagella operate in the Na^+^ energetic cycle mode. Our research may shed light on the energetic reason for how these bacteria are confined to a narrow chemocline zone and thrive in the extreme conditions of soda lakes.

## 1. Introduction

Recent studies of new extreme habitats of living organisms have led to the discovery of new subdivisions of bacteria and archaea, and significantly expanded knowledge of the possible limits of life [1,2]. Following this, new data on the energetics [3,4,5,6,7,8,9,10,11,12,13] and biocatalysis [14,15,16] in organisms capable of living in extreme conditions have been disclosed. Soda lakes belong to such areas, being characterized by a combination of several extremes [17]. Bacteria of the genus *Thioalkalivibrio*—belonging to the class Gammaproteobacteria, and distantly related to anoxygenic purple sulfur bacteria of the genus *Ectothiorhodospira*—are common inhabitants of soda lakes [18,19,20,21,22,23,24,25], and belong to the double-extremophilic bacteria [26]. Among them is the strain *Thioalkalivibrio* *versutus*, which has adapted to thriving in soda brines characterized by both strong alkalinity (pH up to 11) and high salinity, with almost saturating salt concentrations (up to 4 M Na^+^) [27]. The strain is obligately aerobic, as described earlier. A surprising adaptation of *Thioalkalivibrio* strains to life in extreme alkaline conditions with saturating sodium concentrations is the use of a sodium pump at the terminus of their respiratory chain (Na^+^-motive cytochrome-*c* oxidase) [28,29]. This is the first Na^+^-motive oxygen-reducing enzyme proven to operate in living organisms. Long before the discovery of these bacteria, the emergence of such a sodium pump was predicted to counteract the low proton-motive force (Δ*p*) on membranes of alkaliphiles in alkaline media [30,31,32], but only 30 years later was the sodium pump discovered [29]. Our data showed that, indeed, in *T. versutus* cells the Δ*p* is as low as −80 mV [33]. According to Guffanti and Krulwich, as was shown in alkaliphilic *Bacillus* strains, this value is not enough to drive ATP synthesis [34,35]. At the same time, in *T. versutus*, operation of the Na^+^-motive cytochrome-*c* oxidase enables the maintenance of a high negative electrical potential on their membranes [33], which is even higher than that shown in many alkaliphiles [35]. Direct methods have shown that the described sodium pump generates a high sodium-motive force (Δ*s* [36]) across the bacterial cytoplasmic membranes [29], and has properties inherent in alkaliphilic proteins [37].

According to the genomic databases, in *Thioalkalivibrio* representatives, a possible presence of Δ*s* consumers can be predicted; among such Δ*s* consumers are secondary Na^+^-transporters and Na^+^-type flagella [38]. However, in *T. versutus*, the functioning of the sodium energetic cycle composed of a Δ*s* generator and a Δ*s* consumer has not been experimentally proven. Previously, we have undertaken the first steps towards achieving bacterial motility [39]. In the present study, we describe the modified procedure for the selection of stable motile bacterial cells suitable for the study of bioenergetic characteristics and, using inhibitor analysis, experimentally ascertain the Na^+^ type of the flagellar motor. Our findings allow us to verify in *T. versutus* the functioning of the sodium energetic cycle, which consists of a Δ*s* generator and a Δ*s* consumer.

## 2. Results and Discussion

**Figure 1:**
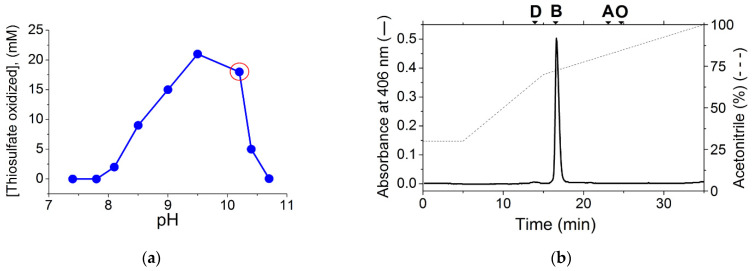
*Thioalkalivibrioversutus* batch culture characteristics: (**a**) pH profile for substrate consumption by *T. versutus* measured in batch culture. Each point corresponds to independent cultivation at constant pH. The red circle depicts cultivation conditions used in the study, and for the isolation of *T. versutus* from a swimming culture. (**b**) Heme profile of the *T. versutus* membranes, isolated from the cells cultured at pH 10.2 under oxygen-limited conditions. A ruler with the elution times for standard heme D, B, A, and O is shown at the top of the graph. Elution was carried out with a gradient of acetonitrile in water containing 0.05% trifluoroacetic acid (dashed line).

Previously, heme of types B, D, and O was found in the cells of several different *Thioalkalivibrio* strains [40,41], indicating the possibility of induction of different types of oxygen reductase enzymes in representatives of this genus under different conditions. However, the membranes of the *Thioalkalivibrio versutus* strain grown in this work at the optimal pH (pH 10.2) (Figure 1a) contained only cytochromes *c* and *b*—detected spectrally as published earlier [29]—and heme B, according to the HPLC analysis (Figure 1b). Thus, the heme and cytochrome composition of the membranes, as well as the presence of only Na^+^-dependent components on respiration curves in the pH range 7.5–10.5, similar to that shown earlier (see Figure 1 in [29]), indicated that under the conditions used the previously described Na^+^-motive *cbb*_3_ oxidase [29] served as the main terminal oxidase in the strain.

**Figure 2:**
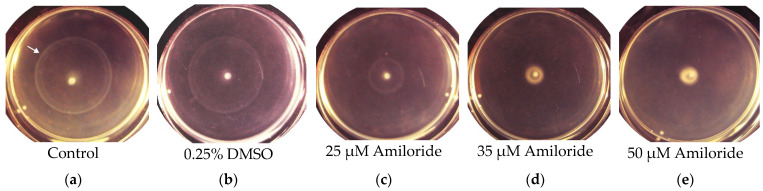
Formation of the bacterial swimming rings by the passaged *T. versutus* cells: (**a**–**e**) *T. versutus* formed bacterial swimming rings (white arrow indicates such a ring on the left panel), seen as radially widening whitish opalescent circles on the surface of the semi-liquid medium in Petri dishes (the photos demonstrate a typical picture 23 h after inoculation). Visible as bright white dots in the center of each Petri dish are unpassaged cells (for details, see Section 3).

The *T. versutus* strain is obligately aerobic, and can derive energy from aerobic oxidation of reduced sulfur compounds [27]. In our experiments, thiosulfate was used as an energy source. To activate motility of the cells that were initially motionless, we used a semi-liquid medium containing 0.3% agarose, placed in Petri dishes. Some time after planting the inoculum in the center of the Petri dishes, expanding whitish swimming rings appeared on the surface of the medium (Figure 2a–c).

**Figure 3:**
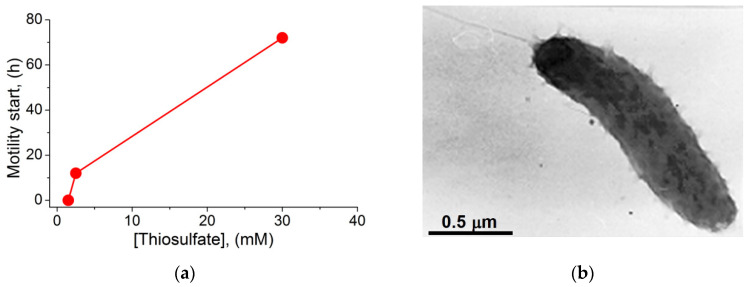
Activation of *T. versutus* cell motility: (**a**) Dependence of the start of motility on the concentration of the growth substrate. (**b**) Electron micrograph of a flagellum-bearing *T. versutus* cell stained with 1% uranyl acetate.

Microscopic examination showed that the cells taken from the swimming rings became motile. The ability of *T. versutus* cells to acquire motility in a semi-liquid medium depended on the thiosulfate concentration in the medium (Figure 3a). The absence of a lag phase before the start of movement was observed at 1.5 mM thiosulfate. An increase in the thiosulfate concentration to 2.5 mM and 30 mM led to a progressive delay in the ability of cells to move. Motile cells sampled from the periphery of the rings had monopolar flagellation and a single flagellum (Figure 3b).

**Figure 4:**
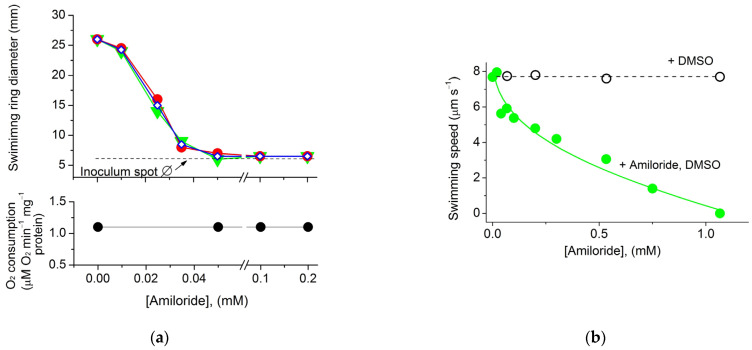
Effect of amiloride on the motility of *T. versutus* cells: (**a**) Dependence of the swimming rings’ diameter (upper panel) and cell respiratory rate (lower panel) on amiloride concentration. For motility, a semi-liquid medium supplied with 1.5 mM thiosulfate as an energy substrate was used. The diameters shown in the graph were recorded 19 h after the start of three independent experiments. The results of each experiment are marked with closed red circles, closed green triangles, or open blue diamonds. At the bottom of the upper panel, the level of inoculum spots’ diameter is indicated (dashed line). (**b**) Dependence of the cell motility speed in a liquid medium on amiloride concentration (closed green circles). The cell motility was examined in a Goryaev chamber, using transmitted light microscopy. The liquid medium for motility experiments contained 100 mM Caps-KOH (pH 9.2), 0.6 M KCl, 10 mM NaCl, and 10 mM ascorbate as an energy substrate. The graph data are the average of three independent experiments. In each independent experiment, the swimming speed value at each indicated concentration of amiloride is the average of 40–45 individual measurements.

To determine the type of flagellar motor, we used amiloride—an inhibitor of Na^+^/H^+^ antiporters and Na^+^ channels of many organisms [42], which is also known as a specific inhibitor of the Na^+^-type flagellar motor [43]. According to Atsumi et al., at alkaline pH, amiloride interfered with the growth of some *Bacillus* and *Vibrio* alkaliphilic species in solid and semi-liquid media [44]. In our experiments, amiloride at concentrations no higher than 50 μM did not affect the growth of *T. versutus* culture, and was used in further experiments at these low concentrations. Testing in semi-liquid media showed that amiloride at low concentrations blocked the expansion of swimming rings (Figure 2c–e). Thus, it could be assumed that *T. versutus* motility is provided by Na^+^-motive flagella. However, it was previously noted that, in contrast to semi-liquid media—in which amiloride in concentrations of tens of micromoles can inhibit bacterial growth—liquid media make it possible to study the effects of amiloride at higher concentrations [44]. For a more detailed study of amiloride’s effects, we used microscopy to analyze the cell motility speed in a liquid medium in a Goryaev chamber. For this, the cells selected for maximal swimming speed in a semi-liquid medium were collected and studied when the substrate was washed out. The motility was initiated by adding thiosulfate (Figure 4a) or a cytochrome oxidase substrate (ascorbate) (Figure 4b). Thiosulfate-initiated cell respiration remained constant with increasing amiloride concentration (Figure 4a, lower panel); this indicates that amiloride had no effect on the oxygen-reducing Δ*s* generator cytochrome oxidase. On the other hand, in both semi-liquid (Figure 4a, upper panel) and liquid media (Figure 4b), amiloride suppressed bacterial motility, albeit with different efficiency. While the half-maximal inhibitory effect was comparable in both variants—I_50_ 25 μM and 30 μM, respectively—complete inhibition of motility in a semi-liquid medium was achieved at a significantly lower concentration of amiloride (50 μM, versus almost 1 mM in a liquid medium). In both cases, dimethyl sulfoxide (DMSO) free of amiloride (used as a solvent for amiloride in other experiments) did not affect motility (Figure 2b and Figure 4b). The picture of the dependence of *T. versutus* motility on amiloride concentration in a liquid medium coincides with the previously published data for *Vibrio alginolyticus* [45], and confirms that the cells use the sodium flagellar motor.

**Figure 5:**
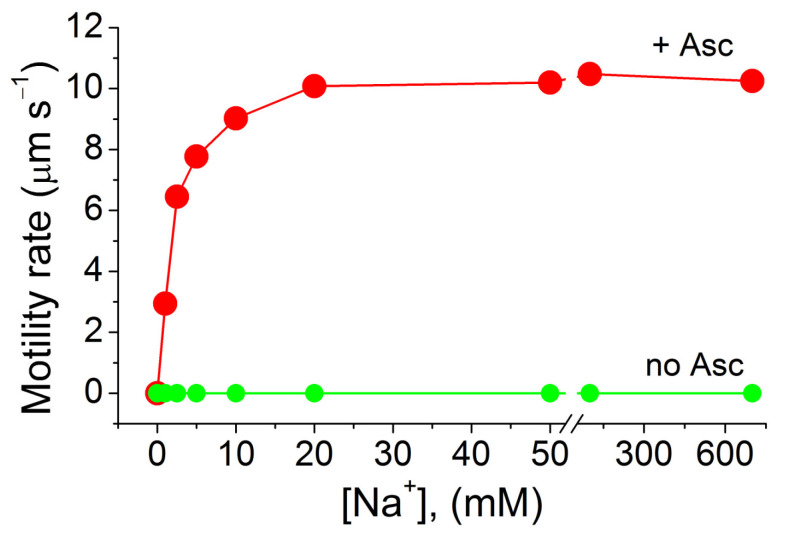
Motility recovery of the initially motionless *T. versutus* cells in a liquid medium; 10 mM potassium ascorbate was used as a substrate. Concentration of sodium in the bacterial suspension was varied by the addition of small aliquots of the incubation medium, containing 4 M NaCl (for details, see Section 3).

To verify the effect of sodium ions on cell motility, we used bacterial cells washed to remove sodium and then placed in a liquid medium free of sodium and respiratory substrates. After one day of starvation, such bacterial cells became motionless. The motility of the cells was recovered upon the addition of NaCl, achieving final concentrations of 1–20 mM in the presence of ascorbate in the medium (Figure 5). The effect of sodium was specific to monovalent cations, since neither potassium chloride nor lithium chloride restored bacterial motility—similar to findings for the alkaliphilic *Bacillus* YN-1 [46]. The dependence of the motility and respiratory activity of *T. versutus* cells on sodium concentration, as well as their sodium specificity, proved to be the same, which could indicate that the Na^+^-motive cytochrome oxidase and Na^+^-type flagella operated in one and the same mode of the Na^+^ energetic cycle. Interestingly, the effect of sodium chloride in nearly the same concentration range was observed on the motility of *Vibrio alginolyticus* 1854, *Vibrio cholerae* VIO5 [47], *Vibrio alginolyticus* Nap1 [48], *Vibrio parahaemolyticus* [45], and an alkaliphilic *Bacillus*—*B. firmus* RAB [46].

However, such a flagellar motor could be started either directly by Δ*s*—generated by means of a primary Na^+^ pump, as in *Vibrio* species at alkaline pH [49]—or indirectly, when Δ*p*, generated by a primary H^+^ pump at neutral pH, is converted into Δ*s* by a secondary Na^+^-pumping mechanism—for example, a Na^+^/H^+^-antiporter [49]—as it also takes place in alkaliphilic *Bacillus* species (for review, see [50]). In order to find out what kind of energy transfer mechanism was used by the cells to initiate operation of the flagellar motor, we investigated the effects of protonophores and some inhibitors.

**Figure 6:**
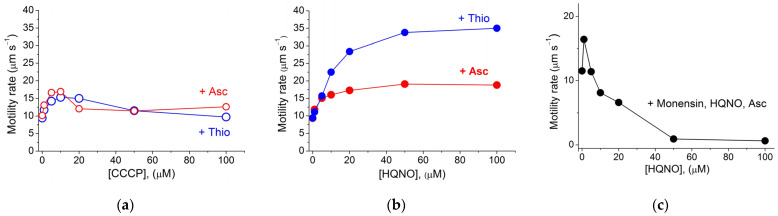
Effects of protonophores CCCP (**a**) and HQNO (**b**), and the Na^+^/H^+^ ionophore monensin (**c**), on bacterial cell motility. (**a**,**b**) Cell motility was started by the addition of either 1.5 mM thiosulfate (blue curves) or (**a**–**c**) 10 mM ascorbate (red curves) in the presence of 10 mM NaCl (final concentrations indicated); (**c**) 50 µM monensin was added to the cell suspension supplied with 10 mM potassium ascorbate in the presence of 10 mM NaCl and the protonophore HQNO (black circles).

Bacterial motility was preliminarily initiated by adding either thiosulfate or ascorbate to the suspension of starved motionless cells in the presence of Na^+^ ions. Figure 6a,b show the effects of the protonophores carbonyl cyanide m-chlorophenylhydrazone (CCCP) and 2-heptyl-4-hydroxyquinoline N-oxide (HQNO) on *T. versutus* motility. It can be seen that CCCP had almost no effect, but HQNO increased motility quite strongly.

Apparently, the favorable effect of HQNO on the motility of the studied bacteria may be explained by the decrease in the electric potential (Δψ) on bacterial membranes due to the protonophorous action of HQNO. As we have shown previously, HQNO, in contrast to CCCP, is an effective protonophore at alkaline pH [29]; this is why HQNO can transport H^+^ from the outer medium to the negatively charged interior of bacteria; as a result, Δψ decreases. This situation may be similar to the state of bacteria with a decrease in energy resources, when cells became motile (Figure 3a). It is likely that, both in the presence of HQNO and with a deficit of energy resources, a decrease in Δψ serves as a signal for the acquisition of motility and the following migration of bacteria to more favorable conditions.

Another possibility arises due to the fact that, according to our data [33], the Δψ level in respiring *T. versutus* can reach a value of −228 mV, which is higher than in other bacteria and mitochondria. High Δψ is dangerous for biomembranes—especially for mitochondrial membranes and mitochondrial metabolism—as has been shown using planar membranes [51]. It cannot be ruled out that a certain high Δψ level in bacterial membranes may be harmful due to increase in membrane permeability for Na^+^, which is present at a very high concentration in the medium.

In the presence of the protonophore HQNO, not only does Δψ decrease, but Δ*p* dissipates, and should an H^+^ pump be involved in the energization of the flagellar motor, the motor would have to stop. However, this did not happen (Figure 6b); on the contrary, the rate of cell motility increased 2–3.5 times in the presence of HQNO, which excludes the secondary mechanism of energization of the flagellar motor when an H^+^ pump and Na^+^/H^+^ antiporter are involved. It should be noted that in the first minutes upon the addition of the respiratory substrate and HQNO to *T. versutus* cells, when Δψ and Δ*p* fall to values close to zero, a 10-fold gradient of sodium ions is generated, corresponding to a very high ΔpNa value of −220–−260 mV (see Figure 3A in [29]). The observed scenario most closely matches the direct use of Δ*s*.

The above effect can be explained by the fact that the protonophore provides an entry of H^+^ counterions into the cell in accordance with the law of electroneutrality, and thereby prevents the electric field from controlling the operation of the Na^+^ pump. As a result, the activity of the primary Na^+^ pump increases, followed by an increase in ΔpNa with the simultaneous dissipation of the electric potential on the membrane. The stimulation effect of protonophore uncouplers on Na^+^ pumping has been described for a number of Δ*s*-generating proteins [52], among which are Na^+^-pumping NADH-quinone oxidoreductases [53], Na^+^-ATPases [54,55,56,57,58], recently described Na^+^-pumping cytochrome oxidase [28,29], and Na^+^-proteorhodopsins [59,60]. Consistent with the above explanation for the effect of HQNO, we showed that in the presence of HQNO, the Na^+^/H^+^ antiporter monensin (dissipating transmembrane [Na^+^] and [H^+^] gradients) abolished the HQNO-induced stimulation of the motility rate (Figure 6c). Thus, the obtained data support the direct use of Δ*s* generated by the primary Na^+^ pump, which could be represented in *T. versutus* by the Na^+^-motive *cbb*_3_ oxidase and/or a Na^+^-motive ATPase.

To test possible mediation of ATPase as a generator of Δ*s*, we used the cells washed to remove sodium and preincubated with or without 20 mM arsenate for a day. The motility of such cells (both in the absence of arsenate when ATPase was functioning, and in the presence of 20 mM arsenate when ATPase was blocked) was successfully triggered by ascorbate in a sodium-dependent mode, and the speed of motility did not depend on the presence of arsenate. These results excluded participation of ATPase in the energization of flagella, and showed that the generation of Δ*s* by the Na^+^-motive cytochrome-*cbb*_3_ oxidase triggers the Na^+^-type flagella motor. Thus, in *T. versutus*, a Na^+^ cycle operates that includes a Δ*s* generator (Na^+^-motive cytochrome-*cbb*_3_ oxidase) and a Δ*s* consumer (a Na^+^-type flagellar motor).

Previously, Chernyak et al. showed that the flagellar motor of marine *V. alginolyticus* is powered by Δ*s* [61]. Later, Atsumi et al. found that, depending on the habitat, *V. parahaemolyticus* can acquire two types of flagellar motor that differ in coupling ions, localization, and functional specialization—namely, laterally located H^+^-motive flagella, and polar-located Na^+^-motive flagella [45]. According to our study, the batch-cultured *T. versutus* strain bears a single polar flagellum, which is clearly a Na^+^-motive flagellar motor.

To our knowledge, here we have demonstrated for the first time that the activation of the Δ*s* generator by protonophores leads to stimulation of the Δ*s* consumer (the Na^+^-motive flagellar motor). In particular, stimulation is manifested to a greater extent in the presence of HQNO, and to a lesser extent in the presence of CCCP, which is consistent with the efficiency of these protonophores at alkaline and weakly acidic/neutral pH, respectively [29]. The reasons why activation of bacterial motility in the presence of protonophores has not been previously described in the literature may be as follows: HQNO inhibits Na^+^-NADH-CoQ-reductase (NQR) [53], the functional operon of which is lacking in the genome of *T. versutus*. Considering the above, it can be assumed that in bacteria bearing NQR the protonophore-stimulating effect of HQNO was leveled by its inhibitory counter-effect on NQR activity [45,47], while CCCP was less effective as a protonophore at alkaline pH.

Being similar in architecture to the known *cbb*_3_ oxidases, the Na^+^-motive oxidase has unique properties that open the way for favorable existence of natronophilic *T. versutus* in soda lakes [37]. Having specific motifs in the amino acid sequence of the catalytic subunit capable of binding Na^+^ ions, this CO shows a strongly negative redox potential of the cytochrome-*c*-bearing subunits, which apparently promote energy conversion under alkaline conditions [37]. Based on our previous studies of *T. versutus* cells, we can deduce the Δ*s* value generated on their membranes in 5 min after substrate addition. The Δ*s* is composed of Δψ (−228 mV) [33] and 60ΔpNa (−42 mV) [29], resulting in an overall −270 mV. It can be assumed that the previously demonstrated much lower ^22^Na^+^ leakage across membranes in *T. versutus* compared to non-extremophilic *Paracoccus denitrificans* (see Figure 3 in [29]) ensures the maintenance of such a high Δ*s* value. This high value helps explain how extreme bacteria not only escape depression as a consequence of low Δ*p*, but flourish and offset the much higher energy costs they incur to survive in severe extreme conditions.

The obtained data may also explain the ecological preferences of *T. versutus*. It is known that these bacteria are confined to living in a narrow zone called the chemocline. The sodium energetic cycle, formed by Na^+^-motive cytochrome oxidase and Na^+^-type flagella, could serve as a mechanism by which the bacteria are retained in this zone in the presence of sodium and a highly alkaline pH of the medium. Obviously, far from the chemocline zone, the concentration of sulfur-containing substrates decreases and, according to our results, activates the bacterial motility providing them with access to areas more rich in nutrients. On the other hand, when bacteria approach the chemocline zone, which is rich in sulfur-containing substrates, the speed of their motility should slow down, leading to the retention of bacterial cells in this zone.

## 3. Materials and Methods

### 3.1. Materials

Amiloride, monensin, carbonyl cyanide m-chlorophenylhydrazone (CCCP), 2-heptyl-4-hydroxyquinoline N-oxide (NQNO), KCl and KOH free of sodium were purchased from Sigma-Aldrich (St. Louis, USA); dimethyl sulfoxide (DMSO) was purchased from Merck (Darmstadt, Germany).

### 3.2. Bacteria Growth Conditions for the Experiments

The type strain *Thioalkalivibrio versutus* AL2 (*T. versutus*) was cultured in 25 mL of a “soda” medium at pH 10.2, with thiosulfate as a growth substrate [27], under conditions of limited aeration in 50 mL Falcon tubes on a Biosan ES-20 rotary shaker (Biosan, Riga, Latvia) at 90 rpm and 30 °C until the stationary phase of growth. During that time, the tubes were set at an angle of 120° relative to the horizon. The content of thiosulfate consumed in the nutrient medium was estimated by the cyanolytic method, which allows for individual quantitative determination of thiosulfate, tetrathionate, and trithionate [62].

### 3.3. Analysis of Hemes

The membranes of the grown bacterial cells were isolated as described previously [63], with some modifications [37], and analyzed for heme content. For this purpose, heme was extracted from the isolated cellular membranes, and the following heme analysis was performed using HPLC as described previously [64], with some modifications [65]. Heme was extracted and separated via reversed-phase chromatography on a Diasorb-C16 column 3 × 250 mm (Elsico, Moscow, Russia) at a flow rate of 1 mL/min, with detection at 406 nm on a PU 4110 chromatograph (Philips Scientific (Pye Unicam), Cambridge, UK). To calibrate the chromatographic column, the elution times of standard heme A and B isolated from bovine heart cytochrome oxidase, and heme D and O isolated from *E. coli*, were determined as described previously [65].

### 3.4. Selection of Motile Cells

For experiments on cell motility, the most motile bacterial cells were selected by multiple passages. For this, the stationary cell culture was sedimented in a benchtop Eppendorf centrifuge 5418 (5000 rpm, 5 min, 20 °C), and a thick bacterial suspension withdrawn from the surface of the pellet (inoculum, 20 μL) was placed in the center of Petri dishes filled with a semi-liquid medium containing 0.3% agarose and the “soda” medium (pH 10.2), supplied with a growth substrate (2.5 mM thiosulfate) as described previously. One minute after placing the inoculum of the passaged cells in the center of Petri dishes, an inoculum of control cells (3 μL) that were not passaged was added (visible as a bright white dot in the center of Petri dishes; Figure 2). To view Petri dishes inoculated with passaged cells only (a total inoculum volume of 23 µL), see Figure S1. Upon inoculation, the agarose plates were left on a flat surface at 20 °C, and after a few hours the cells began to form swimming rings, as shown in Figure 3. The diameter of the swimming rings was measured with a ruler. The cells that ran farthest from the center of the Petri dish were collected and used for the next passage on a fresh Petri dish. After three successive passages, the most motile cells were selected and used in microscopy experiments. Amiloride (an inhibitor of Na^+^ channels) was used as a solution in dimethyl sulfoxide (DMSO). When used on agarose plates, the following concentrations of amiloride were tested: 0.005 mM, 0.01 mM, 0.02 mM, 0.035 mM, 0.05 mM, 0.1 mM, and 0.2 mM. To achieve the indicated concentrations of amiloride, small aliquots of the stock solution of amiloride in DMSO and, if necessary, DMSO free of amiloride, were added to the cooling agarose medium to maintain a final DMSO concentration of 0.25% (*v*/*v*).

### 3.5. Respiratory Activity

The respiratory activity of the cells was assessed as previously described at 25 °C [29] by monitoring the oxygen consumption of the cell suspension in a thermostatically controlled semi-closed measuring chamber with a useful capacity of 0.75 mL. Oxygen consumption was determined polarographically, using a Clark-type electrode (amperometric oxygen sensor) housed in the measuring chamber and connected to an “Expert” millivoltmeter with a corrosometer function (Eco-Expert, Moscow, Russia). At the output, the millivoltmeter was interfaced with a computer, and the resulting digital signals from the millivoltmeter were continuously recorded in the form of a time-dependent graphic chart using the PC software supplied by the manufacturer.

### 3.6. Evaluation of Swimming Speed in a Liquid Medium

The motility speed was evaluated in a Goryaev chamber filled with a liquid medium of the composition specified in the Results section. The motility observations and speed evaluation were performed using an Eclipse E200 microscope (Nikon, Tokyo, Japan) and a stopwatch. The swimming speeds of the cells were measured for at least 40–45 cells in each experimental condition and then averaged. In experiments to determine the ion dependence of motility, the sodium concentration in the bacterial suspension was varied by adding small aliquots of incubation medium containing 4 M NaCl and an appropriate volume of 4 M KCl solution to maintain a constant ionic strength with each new desired concentration of NaCl. The protonophores CCCP and HQNO, as well as the ionophore monensin, were used as stock solutions in ethanol and added to the bacterial suspension as small aliquots to achieve the required concentrations. The final ethanol concentrations did not exceed 0.5% (*v*/*v*). The effects of each compound—CCCP or HQNO—as well as monensin on the motility rate were determined 1 min after the addition of various concentrations of these ionophores.

### 3.7. Electron Microscopy

For total electron microscopy, the cells were fixed with paraformaldehyde (4% *w*/*v* final) for 1 h at 4 °C, and applied to a copper grid coated with collodium film for 5 min, then stained for 30 s in 1% (*w*/*v*) uranyl acetate. The preparations were observed on a JEOL 100 transmission electron microscope (JEOL Ltd., Tokyo, Japan). 

## 4. Conclusions

Natronophilic *T. versutus* bacteria are motile and equipped with polar flagella of the Na^+^ type. The energetic strategy of the bacteria when they perform mechanical work such as motility is to use the Na^+^ cycle, which includes operation of the Na^+^-motive *cbb*_3_ oxidase and Na^+^-type flagella. The results are consistent with the hypothesis that Na^+^-motive motility is an adaptive function in these bacteria that, under conditions of extreme alkalinity and salinity, allows them to stay in a zone enriched with sulfur-containing nutrients.

## Supplementary Materials

The following are available online at https://www.mdpi.com/article/10.3390/ijms23041965/s1.
