# Supplementary material for: Sodium Energetic Cycle in the Natronophilic Bacterium Thioalkalivibrio versutus
Source: Int J Mol Sci. 2022 Feb 10;23(4):1965. doi: 10.3390/ijms23041965 (PMC8874543; doi:10.3390/ijms23041965)
Supplement: Supplementary file 1 [file ijms-23-01965-s001.zip › ijms-1561468-supplementary.pdf]

Supplementary Materials

Sodium Energetic Cycle in the Natronophilic Bacteria *Thioalkalivibrio versutus*

Maria S. Muntyan[1,]*, Mikhail B. Viryasov[1], Dimitry Y. Sorokin[2,3] and Vladimir P. Skulachev[1]

[1] Belozersky Institute of Physico-Chemical Biology, Lomonosov Moscow State University, Leninskie gory, 119991 Moscow, Russian Federation; viryasov@genebee.msu.ru; skulach@genebee.msu.ru
[2] Winogradsky Institute of Microbiology, Federal Research Centre of Biotechnology, Russian Academy of Sciences, 117312 Moscow, Russian Federation; soroc@inmi.ru
[3] Department of Biotechnology, Delft University of Technology, 2628 BC Delft, The Netherlands
* Correspondence: muntyan@genebee.msu.ru

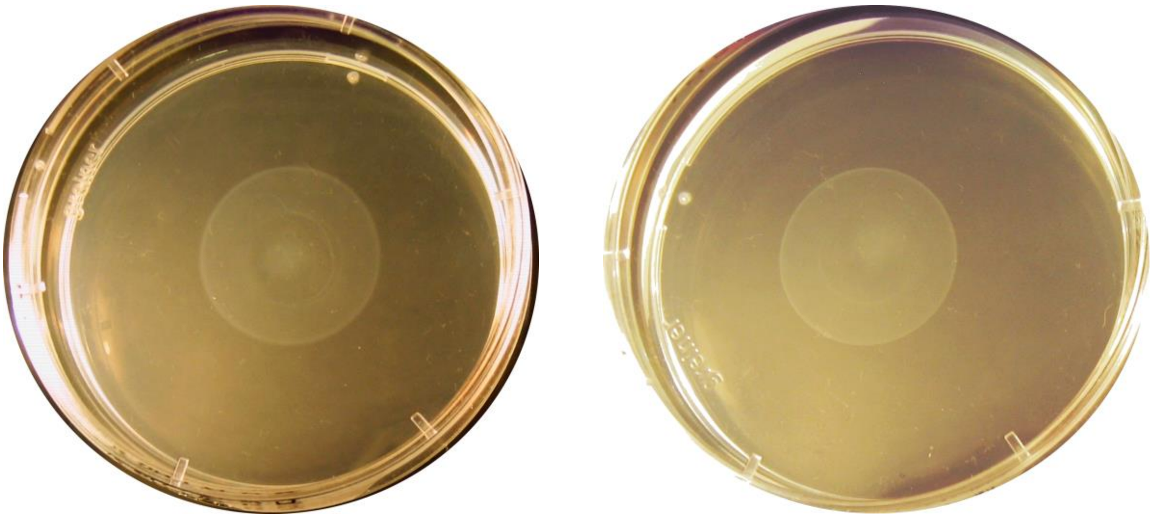

(**a**) (**b**)

Figure S1. Formation of the bacterial swimming rings by the passaged *Thioalkalivibrio versutus* cells 19 h after inoculation: (**a**,**b**), Two different Petri dishes inoculated with the passaged bacterial cells only are shown (for details, see "Materials and Methods").
